# Phenotypic and Genotypic Identification of Bacteria Isolated From Traditionally Prepared Dry Starters of the Eastern Himalayas

**DOI:** 10.3389/fmicb.2019.02526

**Published:** 2019-11-05

**Authors:** Pooja Pradhan, Jyoti Prakash Tamang

**Affiliations:** DAICENTRE (DBT-AIST International Centre for Translational and Environmental Research) and Bioinformatics Centre, Department of Microbiology, School of Life Sciences, Sikkim University, Gangtok, India

**Keywords:** Eastern Himalayas, starters, 16S rRNA sequencing, bacterial diversity, lactic acid bacteria

## Abstract

Preparation of dry starters for alcohol production is an age-old traditional technology in the Eastern Himalayan regions of east Nepal, the Darjeeling hills, Sikkim, and Arunachal Pradesh in India, and Bhutan. We studied the bacterial diversity in 35 samples of traditionally prepared dry starters, represented by *marcha* of Nepal, Sikkim, the Darjeeling hills, and Bhutan, *phab* of Bhutan, and *paa, pee*, and *phut* of Arunachal Pradesh, respectively. Populations of bacteria in these starters were 10^5^ to 10^8^ cfu/g. A total of 201 bacterial strains were isolated from starter samples, phenotypically characterized, and their identities confirmed by the 16S rRNA sanger sequencing method. The dominant phylum was *Firmicutes* (85%), followed by *Proteobacteria* (9%), and *Actinobacteria* (6%). Lactic acid bacteria (LAB) (59%) formed the most abundant group, followed by non-LAB (32%) and Gram-negative bacteria (9%). Based on the 16S rRNA gene sequencing result, we identified LAB: *Enterococcus durans, E. faecium, E. fecalis, E. hirae, E. lactis, Pediococcus acidilactici, P. pentosaceus, Lactobacillus plantarum* subsp. *plantarum, Lb. pentosus, Leuconostoc mesenteroides*, and *Weissella cibaria*; non-LAB: *Bacillus subtilis* subsp. *inaquosorum, B. circulans, B. albus, B. cereus, B. nakamurai, B. nitratireducens, B. pseudomycoides, B. zhangzhouensis, Kocuria rosea, Staphylococcus hominis* subsp. *hominis, S. warneri, S. gallinarum, S. sciuri, Lysinibacillus boronitolerans, Brevibacterium frigoritolerans*, and *Micrococcus yunnanensis*; Gram-negative bacteria: *Pseudomonas putida, Klebsiella pneumoniae, Enterobacter hormaechei* subsp. *xiangfangensis, E. hormaechei* subsp. *steigerwaltii*, and *Stenotrophomonas maltophilia*. We characterized diversity indexes of the bacterial community present in traditionally prepared dry starters. This is the first report on the bacterial diversity of traditionally dry starters of the Eastern Himalayas by sanger sequencing.

## Introduction

The Himalayas, well known for high mountains with natural beauty and rich biological resources, extend from peak Nanga Parbat in Pakistan to peak Namcha Barwa across India, Nepal, and Bhutan (Le Fort, 1975). Based on geo-morphology and demography, the Himalayas are divided into three regions, the Western, Central, and Eastern Himalayas (Nandy et al., 2006). The geographical location of the Eastern Himalayas extends from eastern Nepal, North East India (Darjeeling hills, Sikkim, and Arunachal Pradesh), Bhutan, and Tibet Autonomous Regions in China (Saha, 2013). Agrarian and pastoral types of mountain farming dominate the agriculture and animal husbandry systems in the Eastern Himalayas, and these are practiced by diverse ethnic communities (Sharma et al., 2007; Bhasin, 2013). Many major and rare types of ethnic fermented foods and beverages are traditionally produced from locally available plant and animal resources and are made into a wide variety of flavorsome cuisine that is consumed as staple diets, side-dishes, curries, soups, condiments, and alcoholic drinks by ethnic people of the Eastern Himalayas (Tamang, 2010; Tamang et al., 2012). The majority of ethnic Himalayan people drink home-made traditional alcoholic beverages and distilled liquor prepared from cereals (rice, finger millets, and maize) as per socio-compulsion but also for enjoyment. Vinification, malting, and brewing processes for alcohol production are completely unknown in the food culture of the Himalayan people; instead, rice or finger millets are fermented into mildly alcoholic (∼4%) beverages (Thapa and Tamang, 2004) by using dry starters, which are unique to these regions.

The Himalayan people have been practicing the art of starter-making using indigenous technology for centuries by using overnight-soaked and pounded rice flours mixed with wild herbs, spices, and 1–2% of previously prepared dry starters in powder form to make doughs. Doughs mixtures with desirable shapes and sizes are placed in fresh fern leaves and allowed to ferment for 2–3 days at room temperature, and the freshly fermented doughs are then sun dried for 2–3 days to get dry starters (Thakur et al., 2015; Anupma et al., 2018). Every ethnic community in the Western, Central, and Eastern Himalayas prepare amylase and alcohol-producing starters with slight variation in the use of substrates, such as rice or wheat, and wrapping materials, such as fern fronds, paddy straw, or plant leaves. In local languages, these are termed *marcha* in Nepal, the Darjeeling hills, and Sikkim in India (Shrivastava et al., 2012; Thakur et al., 2015; Anupma et al., 2018), *mana* and *manapu* in Nepal (Nikkuni et al., 1996), *phab* in Bhutan (Tamang, 2010), *chowan* in Tripura, *dawdim* in Mizoram, *humao, modor pitha* in Assam, *hamei* in Manipur, *khekhrii* in Nagaland, and *phut* in Arunachal Pradesh (Anupma et al., 2018) in India. Similar types of alcohol-producing starters are also prepared in South East Asia by ethnic Asian communities, such as the Vietnamese *benh* (Dung et al., 2007), Korean *nuruk* (Jung et al., 2012), Indonesian *ragi* (Surono, 2016), Philippine *bubod* (Kozaki and Uchimura, 1990), Chinese *daque* or *chiu* o*r chu* (Chen et al., 2014), Thai *loogpang* (Limtong et al., 2002), and Cambodian *dombea* (Ly et al., 2018). The most remarkable advent in the traditional preparation of starter cultures is the practice of the “back-slopping method” (terminology in modern food microbiology) used by ethnic Asians irrespective of their geographical locations for sub-culturing the desirable and essential microbiota.

**FIGURE 1:**
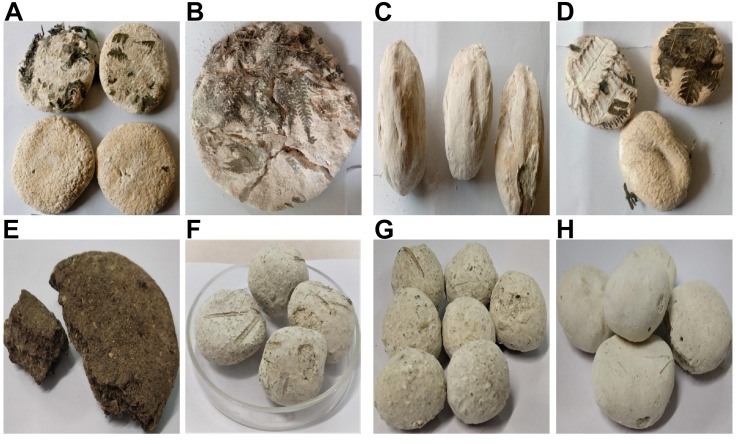
Different types of dry starters from the Eastern Himalayas: **(A)** *Marcha* from Nepal, **(B)** *Marcha* from Darjeeling, **(C)** *Marcha* from Sikkim, **(D)** *Marcha* from Bhutan, **(E)** *Phab* from Bhutan, **(F)** *Paa* from Arunachal Pradesh, **(G)** *Pee* from Arunachal Pradesh, and **(H)** *Phut* from Arunachal Pradesh.

Traditionally prepared dry starters show coexistence of mixed microbiota represented by different genera and species of filamentous molds (Hesseltine et al., 1988; Tamang et al., 1988; Sha et al., 2019), yeasts (Hesseltine and Kurtzman, 1990; Jeyaram et al., 2008, 2011; Sha et al., 2017, 2018, 2019), and bacteria (Hesseltine and Ray, 1988; Tamang et al., 2007; Sha et al., 2017) for saccharification (Lee and Lee, 2002; Thapa and Tamang, 2004), liquefaction (Pervez et al., 2014), and ethanol production (Tsuyoshi et al., 2005; Zheng et al., 2011) to produce traditional alcoholic beverages and distilled liquor in many South East Asian countries, including Nepal, India, and Bhutan in the Himalayas. Filamentous molds (species of *Rhizopus, Mucor, Aspergillus*), and yeasts (species of *Saccharomyces, Pichia, Sacharomycopsis, Candida*) are involved in saccharification and liquefaction; they produce amylolytic enzymes for degrading starch into sugars, and the main alcohol-producing yeasts are *Saccharomyces* for alcohol production (Nout and Aidoo, 2002; Thapa and Tamang, 2004; Li et al., 2012; Nile, 2015). Besides the saccharifying and alcohol-producing ability of mycelia molds and yeasts, some bacterial species present in starters also contribute by imparting flavor, antagonism, and acidification onto the fermenting substrates (Tamang et al., 2007; Huang et al., 2017). Extensive profiling of the diversity of yeasts and mycelial molds in various traditionally prepared dry starters collected from different places of North East India have been reported earlier (Tamang et al., 1988; Tamang and Sarkar, 1995; Tsuyoshi et al., 2005; Jeyaram et al., 2008, 2011; Bora et al., 2016; Sha et al., 2017, 2018, 2019). Samples of *marcha* collected from the Darjeeling hills and Sikkim were analyzed earlier and reported few species of bacteria: *Pediococcus pentosaceus* (Tamang and Sarkar, 1995), *Pediococcus pentosaceus* and *Lb. brevis* (Tamang et al., 2007), *Acetobacter*, *Fructobacillus, Lactococcus*, *Lactobacillus, Leuconostoc*, *Burkholderia*, and *Gluconacetobacter* (Sha et al., 2017). However, no published reports on bacterial diversity associated with *marcha* in Nepal and Bhutan, *phab* in Bhutan, and *paa, pee*, and *phut* in Arunachal Pradesh are available to date. *Marcha* (Figures 1A–D) is a dry rice-based starter, prepared by the Gorkha/Nepali community in the Darjeeling hills and Sikkim in India, east Nepal, and south Bhutan, to ferment boiled finger-millets into a sweet-sour, mildly alcoholic beverage called *kodo ko jaanr* or *chyang* (Tamang et al., 1996). *Marcha* is prepared from soaked and pounded rice flours mixed with some wild herbs, few spices, 1–2% of previously prepared powdered *marcha* by the back-slopping method to make doughs that are placed in fresh ferns leaves, are allowed to ferment for 2–3 days, and are then sun dried for 2–3 days to get dry starters. *Phab* or *pho* (Figure 1E) is a dark brown, flattened, cake-like starter prepared from powdered maize by the Drukpa community in Bhutan to produce a home-made distilled alcoholic drink called *ara* from barley and finger millets (Anupma et al., 2018). *Paa* (Figure 1F), *pee* (Figure 1G), and *phut* (Figure 1H) are dry starters prepared from rice by the Nyshing, Apatani, and Mongpa communities of Arunachal Pradesh, respectively (Anupma et al., 2018). *Pee* is used to ferment rice into a mildly alcoholic beverage called *opo* by the Nyshing tribes, a mildly alcoholic drink called *apong* by the Apatani, and *phut* is used to prepare a sweet-sour, mildly alcoholic beverage called *themsing* by the Mongpa tribes of Arunachal Pradesh (Shrivastava et al., 2012). Preparation of *marcha, phab, paa, pee*, and *phut* is more or less similar except for some variation in the use of substrates, such as rice in the case of *marcha, phut, paa*, and *pee* and maize-rice husk in *phab*, and wrapping materials, of which fern leaves are used for fermenting rice flour during *marcha* preparation, dry paddy straws are used for *phab* preparation, and locally available plant leaves are used for the preparation of *paa, pee*, and *phut*. We collected dry samples of *marcha, pee, paa, phut*, and *phab* from different places in the Eastern Himalayan regions of Nepal, India, and Bhutan to profile the bacterial diversity as information on yeasts and the mycelial molds community is already available (Sha et al., 2017, 2018, 2019). The present study aimed to profile bacterial diversity isolated from *marcha, pee, paa, phut*, and *phab* based on phenotypic and biochemical tests that use the 16S rRNA gene sequencing method.

## Materials and Methods

### Samples

A total of 35 samples of traditionally prepared dry starters were collected in pre-sterile poly bags from different places located in the Eastern Himalayas viz *marcha* (8 samples) from Nepal, *marcha* (5) from the Darjeeling hills, *marcha* (8) from Sikkim, *marcha* (5) from Bhutan, *paa* (2), *pee* (3), and *phut* (2) from Arunachal Pradesh, and *phab* (2) from Bhutan (Table 1). Collected samples were transported and kept in a desiccator at room temperature since traditionally sun-dried starters are stored in a dry place for more than a year (Tamang et al., 1996).

**TABLE 1:** Bacterial load of dry starters from the Eastern Himalayas.

		**Sample**	**Altitude**			**Moisture**		**cfu/g**
**Sample**	**Region**	**Collection Site**	**(Meter)**	**Latitude**	**Longitude**	**content (%)**	**pH**	**(×10^7^)**
*Marcha*	Nepal	Dharan	371	26°48′ N	87°17′ E	12.5 (9.6−17.0)	5.6 (5.5−5.9)	2.1 (1.1−2.9)
		Dhankuta	1154	26°53′ N	87°8 ′ E			
		Hiley	857	27°02′ N	87°24′ E			
		Hathikharka	1394	27°01′ N	87°32′ E			
*Marcha*	Darjeeling hills	Darjeeling	2059	27°04′ N	88°26′ E	13.1 (12.9−13.3)	5.4 (5.2−5.6)	15.3 (11.0−19.6)
		Kalimpong	1176	27°07′ N	88°47′ E			
*Marcha*	Sikkim	Pakyong	1341	27°24′ N	88°59′ E	11.8 (10.0−13.4)	5.7 (5.6−5.9)	18.5 (10.2−26.5)
		Gangtok	1637	27°32′ N	88°61′E			
		Recab	1072	27°21′ N	88°50′E			
		Basilakha	906	27°22′ N	88°60′ E			
*Marcha*	Bhutan	Gedumari	1045	26°90′ N	89°39′E	13.76 (11.8−15.72)	5.7 (5.5−5.9)	0.01 (0.01−0.02)
		Thimphu	2401	27°47′ N	89°62′ E			
*Paa*	Arunachal Pradesh	Lower Subansiri	661	27°8′ N	93°6′E	11.7 (11−12)	5.1 (5−5.2)	2.3 (2.0−2.6)
*Pee*		Ziro valley	1576	27°53′ N	93°81′E	12.1 (11−13)	5.5 (5.2−5.8)	17.6 (16.8−18.4)
*Phut*		Upper Subansiri	1816	28°3′ N	94°E	11.6 (11.4−11.8)	5.2 (5.1−5.3)	11.5 (9.8−13.2)
*Phab*	Bhutan	Dhonakha	2311	27°66′ N	89°70′ E	6.17 (6.13−6.2)	5.2 (5.0−5.4)	0.03 (0.02−0.04)

### Analysis of Moisture and pH

The moisture content of the samples was estimated by a moisture analyzer (OHAUS/MB-45, United States). The pH of the samples was determined by homogenizing 1 g of sample in 10 mL of distilled water, and the readings were taken using a digital pH-meter (Orion 910003, Thermo Fisher Scientific, United States).

### Microbiological Analysis

Dry starter samples were taken from a desiccator, coarsely crushed by a sterile spatula, and 10 g of the powered sample was then homogenized with 90 mL of 0.85% physiological saline in a stomacher lab blender 40 (Seward, United Kingdom) for 2 min. The homogenized samples were serially diluted in the same diluents, and 1 mL of appropriate diluents was then plated using specific media by the pour plate method. Nutrient agar (MM102, HiMedia, Mumbai, India) for aerobic mesophilic bacterial count, MRS (Man-Rogosa-Sharpe) agar (M641, HiMedia, Mumbai, India) and M17 Agar Base (M929, HiMedia, Mumbai, India) for lactic acid bacteria (LAB), and VRBGA (violet red bile glucose agar) (M581, HiMedia, Mumbai, India) for Gram-negative bacteria were used for the enumeration of bacteria in respective plates. Nutrient agar plates and VRBGA plates were incubated at 37°C for 24 h, and MRS plates and M17 plates were incubated at 30°C for 24–48 h aerobically. The number of colonies was counted as colony forming unit cfu/g. The purity of colonies was maintained by re-streaking them into fresh medium, and this was further confirmed by microscopic examination. The pure colonies were then preserved in 50% glycerol at −20°C for further identification and analysis.

### Phenotypic and Biochemical Characterization

Bacterial isolates were phenotypically characterized for their presumptive identification, and groupings were done on the basis of cell morphology, Gram’s reaction, colony morphology, catalase test, sporulation tests, gas production from glucose, and ammonia production from arginine (Holt et al., 1994). The physiological tests including growth at different pHs, temperatures, and salt tolerance were performed (Tamang et al., 2007). Biochemical characterization of isolates such as sugar fermentation tests, IMViC (Indole, Methyl red; Voges-Proskauer and Citrate) tests specifically for Gram-negative isolates, nitrate reduction tests, and urease tests were also performed using the method of Hammes and Hertel (2003).

### Genotypic Characterization

#### Genomic DNA Extraction

The genomic DNA of each bacterial isolate was extracted by the standard phenol/chloroform method of Cheng and Jiang (2006) with slight modifications. A total of 1 ml of culture grown overnight in MRS broth (M369, HiMedia, Mumbai, India) at 30°C was centrifuged at 8,000 rpm for 10 min. The pellets were centrifuged at 3,000 rpm, suspended in 40 μl 1× TE buffer, and freshly prepared 15 μl lysozyme and 15 μl RNAse enzyme were added to the pellets and incubated at 37°C for 3 h. After incubation, 15 μl of 20% SDS (sodium dodecyl sulfate) and 15 μl of proteinase-K were added and further incubated at 55°C for 3 h. An equal volume of phenol-chloroform solution (49:48) was added to the above mixture, centrifuged at 10,000 rpm for 15 min, and the aqueous upper layer formed was transferred to a fresh vial containing chloroform-isoamyl solution (48:1). It was centrifuged again at 10,000 rpm for 15 min, and the upper aqueous layer formed was transferred to a fresh vial containing 15 μl of 3M sodium acetate and 400 μl of cold absolute alcohol and kept at −20°C for 1 h. The mixture was again centrifuged at 10,000 rpm for 30 min, and the pellets were washed with 70% ethanol and further centrifuged at 10,000 rpm for 30 min. The pellets were then collected, air dried, and suspended in 30 μl 1× TE buffer and stored at −20°C for further analysis. The quality of the genomic DNA was checked by electrophoresis in 0.8% agarose gel and quantified using a NanoDrop spectrometer (ND-1000 spectrometer, NanoDrop technologies, Willington, CT, United States) (Kumbhare et al., 2015).

#### PCR Amplification

The PCR of the 16S rRNA gene from the isolated genomic DNA was amplified using a universal oligonucleotide primer pair 27F (5′-AGAGTTTGATCCTGGCTCAG-3′) and 1492R (5′-TACGGTTACCTTGTTACGACTT-3′) (Lane, 1991) in a Thermal cycler (Applied Biosystems-2720, United States). The reaction mixture, conditions, and protocol for the polymerase chain reaction amplification were performed following the method of Chagnaud et al. (2001). PCR amplification was performed in a mixture containing a final volume of 50 μl of Go green Taq master mix (1×) (NEB), 10 μM of F primer, 10 μM of R primer, and nuclease-free water (NEB). The PCR reaction program was set under the following PCR conditions: 94^°^C for 10 min; 94°C for 1 min, 65°C for 1 min, 72°C for 30 s for 35 cycles, and 72°C for 7 min. PCR products were detected by electrophoresis using 1% agarose, and the bands were stained with 7 μl/100 mL of ethidium bromide (RM813, HiMedia, Mumbai, India) and visualized in UV source Gel-Doc 1000 (Bio-Rad, 97-0186-02, United States). A standard 100 base pair DNA ladder (HiMedia, Mumbai, India) was used for the verification of amplicon size.

#### Purification of the PCR Amplicons

The amplified PCR products were then purified using PEG (polyethylene glycol)-NaCl (sodium chloride) precipitation (20% w/v of PEG, 2.5 M NaCl) precipitation method with little modifications of method described by Schmitz and Riesner (2006). About 0.6 volume of 20% PEG-NaCl was added to final volume of PCR products and incubated at 37°C for 30 min. After centrifugation at 12,000 rpm for 30 min, the aqueous solution was discarded, the pellet was washed twice with freshly prepared ethanol (70%) by centrifugation at 12,000 rpm for 30 min. The collected pellet was then air-dried overnight and 20 μl of nuclease-free water was added, and the final purified product was loaded in 1% agarose gel.

#### 16S rRNA Gene Sequencing

PCR products were set up in 5 μl volume for single primer amplification with the same universal primers 27F (5′-AGAGTTTGATCCTGGCTCAG-3′) and 1492R (5′-TACGGTTACCTTGTTACGACTT-3′) (Lane, 1991) for separate reactions for each primer. PCR reaction was set as follows: denaturation for (96°C, 10 s), annealing (50°C, 5 s), and elongation (60°C, 2 min) with a stop reaction at 4°C. The amplicons were then precipitated with 1 μl sodium acetate (3M, pH 5.2) and 24 μl of absolute alcohol, mixed briefly in vortex and incubated at room temperature for 15 min, spun at 12,000 rpm for 20 min, further washed with 70% ethanol, air-dried, and suspended in 10 μl formamide. Sequencing of the amplicons was performed by the Sanger Sequencing method or the Chain-termination DNA (Sanger et al., 1977), the automation of a modified Sanger method that is commonly used to check the sequence of the templates (Heather and Chain, 2016), was carried out in an automated DNA Analyzer (ABI 3730XL Capillary Sequencers, Applied Biosystems, Foster City, CA, United States).

### Bioinformatics

The sequence quality was checked by Sequence Scanner v.1.0 (Applied Biosystems, Foster City, CA, United States). After checking the sequence quality, the sequences were assembled using a ChromasPro 1.5 (McCarty, 1998). The orientation of the assembled sequences was checked using an orientation checker v.1.0. The identity of bacterial isolates was assigned by comparing their DNA sequences with those available in the GenBank NCBI (National Center for Biotechnology Information) database using a BLAST (basic local alignment search tool) 2.0 program (Altschul et al., 1990). The sequences were then aligned by pairwise alignment using clustalW, and the phylogenetic tree was constructed using MEGA7.0 software by the neighbor joining method (Gascuel and Steel, 2006; Kumar et al., 2016). Diversity indices were calculated using a PAST (PAleontological STatistics) v.3.25, which is a comprehensive statistics package used in many fields of life sciences, economics, earth science, engineering, and paleontology (Hammer et al., 2001). The Chao 1 value for species richness was calculated following the method of Chao and Chiu (2016).

### Data Availability

The sequences retrieved from the 16S rRNA sequencing were deposited at GenBank-NCBI under the nucleotide accession number: MK748250-MK748278, MK202997-MK203032, and MK752675-MK752677.

## Results

### Microbial Population

Populations of bacteria in 35 samples of traditionally prepared dry starters collected from different regions of the Eastern Himalayas were 1.0 × 10^5^ to 2.7 × 10^8^ cfu/g (Table 1). The moisture contents of all samples analyzed were 10%–17% except for *phab* of Bhutan in which the moisture content was comparatively low (<6%). Average pH of all samples was 5.5 (Table 1).

### Phenotypic Characterization

We isolated 201 total bacterial isolates from 35 different samples of traditionally prepared starters collected from the Eastern Himalayas, which were represented by 139 isolates from *marcha* (Sikkim 49; Darjeeling 38; Nepal 34, Bhutan 18), 12 isolates from *paa* (Arunachal Pradesh), 17 isolates from *pee* (Arunachal Pradesh), 11 isolates from *phut* (Arunachal Pradesh), and 22 isolates from *phab* (Bhutan). All 201 bacterial isolates were phenotypically characterized based on various biochemical and physiological parameters (Table 2). A total of nine different bacterial genera including unidentified group were presumptively identified based on phenotypic results following Bergey’s manual of bacteriological classification (Holt et al., 1994), which were mostly represented by Gram-positive bacteria (*Pediococcus, Lactobacillus, Enterococcus, Leuconostoc, Bacillus*, and *Staphylococcus*) and two Gram-negative bacteria (*Enterobacter* and *Citrobacter*). We randomly grouped 201 isolates into 68 representative bacterial strains based on phenotypic characterization results (data not shown).

**TABLE 2:** Phenotypic characterization of bacterial isolates from dry starters from the Eastern Himalayas.

**Presumptive Identification (Total number of isolates)**														**Tolerance**								**IMViC test**					
		
	**Sugar fermentation**													**NaCl**		**pH**			**Temperature(^°^C)**			**Indole**	**MR**	**VP**	**Citrate**	**Urease**	**Nitrate**
					
	**Cellobiose**	**Raffinose**	**Sorbitol**	**Arabinose**	**Mellibiose**	**Xylose**	**Lactose**	**Ribose**	**Melizitose**	**Glucose**	**Sucrose**	**Mannitol**	**Rhamnose**	**5%**	**10%**	**3.6**	**9.6**	**10.6**	**15**	**10**	**45**						
*Leuconostoc* (15)	+(6) −(9)	+(7) −(8)	+(5) −(10)	+(4) −(11)	+(9) −(6)	−(10) +(5)	+(9) −(5) v(1)	−(6) +(8) v(1)	+(10) −(5)	+	+(8) −(6) v(1)	+(12) −(3)	+(4) −(10) v(1)	+	+(1) −(14)		+		+(5) −(10)	+(9) −(6)	+(12) −(3)	IMViC test was not determined for Gram-positive bacteria.					
*Enterococcus* (41)	+(18) −(23)	+(29) −(12)	+(23) −(18)	+(14) −(27)	+(34) −(7)	+(18) −(24)	+(34) −(6) v(1)	+(34) −(7)	+(33) −(3) v(5)	+	−(7) +(34)	+(7) −(34)	+(23) −(17) v(1)	+(36) −(5)	+(6) −(30) v(5)	+(6) −(35)	+(35) −(6)		+(13) −(28)	+(32) −(4) v(5)	+(35) −(6)						
*Pediococcus* (57)	+(19) −(38)	+(27) −(30)	+(19) −(38)	+(23) −(26) v(8)	+(28) −(29)	+(23) −(29) v(5)	+(36) −(12) v(9)	+(49) −(5) v(3)	+(32) −(24) v(1)	+	+(33) −(24)	+(13) −(44)	+(38) −(18) v(1)	+(48) v(9)	+(4) −(51) v(2)	+(3) −(51) v(3)	+(40) −(16) v(1)	+(5) −(52)	+(7) −(44) v(6)	+(38) −(13) v(6)	+(46) −(9) v(2)						
*Lactobacillus* (15)	+(12) −(3)	+(5) −(10)	+(10) −(5)	+(5) −(10)	+(10) −(5)	+(5) −(10)	+	+(12) −(3)	+(4) −(11)	+	+(12) −(3)	+(10) −(2) v(3)	+(4) −(11)	+	+(7) −(8)	+(10) −(5)	+(3) −(6) v(6)		+	+(9) −(6)	+(5) −(5) v(5)						
*Bacillus* (21)	+(17) −(4)	+(3) −(18)	+(3) −(18)	+(3) −(18)	+(3) −(18)	+(6) −(15)	+(15) −(6)	+(17) −(4)	+(9) −(12)	+(18) −(3)	−(11) +(10)	+(15) −(6)		+	+(7) −(14)	+(6) −(15)	+(17) v(4)		+	+(19) v(2)	+						
*Staphylococcus* (13)	+(8 −(5)	+(6) −(5) v(2)	+(5) −(8)	+(4) −(9)	+(6) −(7)	+(5) −(6) v(2)	+(9) −(2) v(2)	+(5) −(8)	+(3) −(10)	+	+(8) −(4) v(1)	+(8) −(5)	+(8) −(3) v(2)	+(6) −(2) v(5)	+(2) −(9) v(2)	+(2) −(9) v(2)	+(5) −(7) v(1)		+(2) −(10) v(1)	+(5) −(7) v(1)	+(9) −(3) v(1)						
Unidentified (21)	+(12) −(9)	+(7) −(14)	+(4) −(17)	+(11) −(8) v(2)	+(8) −(13)	+(9) −(10) v(2)	+(12) −(6) v(3)	+(18) −(3)	+(12) −(9)	+	+(11) −(10)	+(3) −(18)	+(12) −(9)	+	+(2) −(19)	+(1) −(19) v(1)	+(14) −(6) v(1)	+(3) −(17) v(1)	+(11) −(8) v(2)	+(15) −(3) v(3)	+(15) −(4) v(2)						
*Enterobacter* (8)	+	−	+(2) −(6)	+	−	+	−	+(6) −(2)	−	+	+	+	+	Physiological tests were not done for Gram–negative bacteria								−	−	+	+	+	+
*Citrobacter* (10)	+(5) −(5)	-	−	+(5) −(5)			+(8) −(2)	+(7) −(3)		+(8) −(2)	−(7) +(3)	−(5) v(5)	−(7) +(3)									−	−	−	+(6) −(4)	−(6) +(4)	+(6) −(4)

*+,Positive; −,Negative; v, Variable; Numbers in parenthesis indicates number of isolates; All strains of Gram-negative bacteria were tested for IMViC either +/−. +,Positive; −,Negative; v, Variable; Numbers in parenthesis indicates number of isolates; All strains of Gram-negative bacteria were tested for IMViC either ±.*

### Molecular Identification of Bacterial Isolates

**FIGURE 2:**
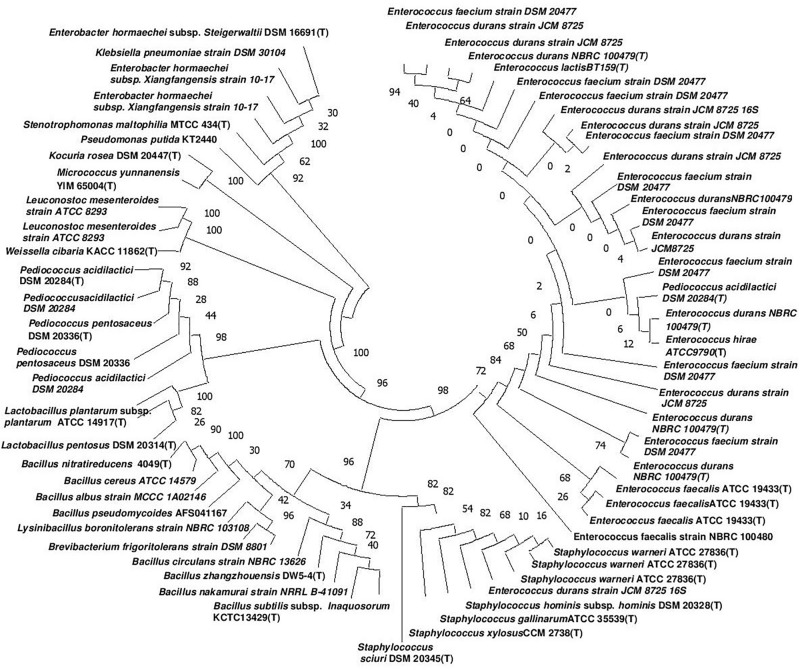
Phylogenetic tree of the nucleotide sequences of 68 bacteria isolates from 35 different samples of dry starter from the Eastern Himalayas based on 16S rRNA sequencing. The tree was constructed by using the Neighbor-joining method (Gascuel and Steel, 2006) with bootstrap values for 1,000 replicates shown at the nodes of the tree using MEGA-7 (Kumar et al., 2016). The optimal tree with the sum of branch length = 0.98855936 is shown. The evolutionary distances were computed by the Maximum Composite Likelihood method (Varin et al., 2011) and are expressed in the units of the number of nucleotide substitutions per site. All positions containing gaps and missing data were eliminated. There were 308 total positions in the final dataset.

**FIGURE 3:**
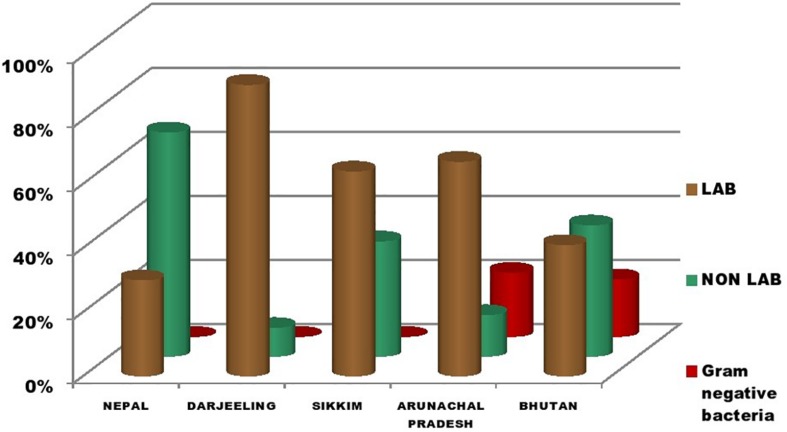
Distribution of LAB, non-LAB, and Gram-negative bacteria in dry starters from the Eastern Himalayas.

**FIGURE 4:**
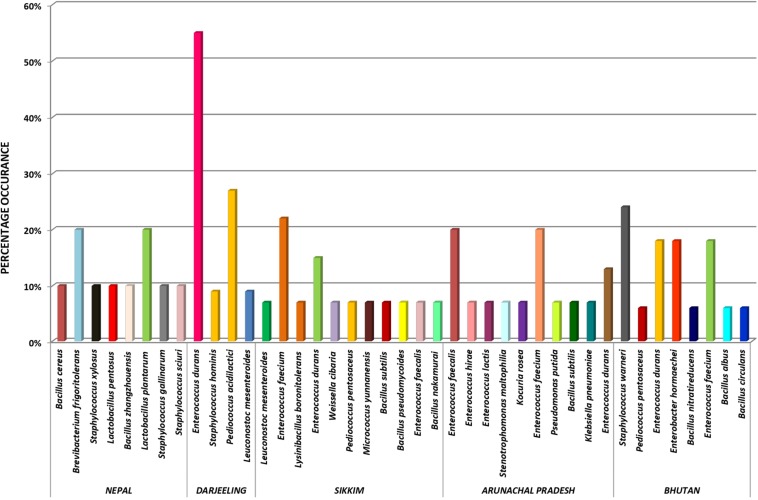
Distribution of bacterial species in dry starters from the Eastern Himalayas.

The genomic DNA of each isolate of all 68 representative bacteria strains was extracted and PCR products were prepared for identification by 16S rRNA gene sequence using the Sanger method. DNA sequences of bacterial isolates were assigned by comparing them with those available in the GenBank NCBI database using a BLAST 2.0 program (Altschul et al., 1990) for identification. The phylogenetic trees of the nucleotide sequences of 68 bacteria isolates from samples of *marcha, paa, pee, phut*, and *phab* were constructed using the Neighbor-joining method with 1,000 bootstrap value replicates (Figure 2). The 16S rRNA sequencing results showed three bacterial phyla represented by *Firmicutes* (85%), *Proteobacteria* (9%), and *Actinobacteria* (6%). The phylum distribution of the *marcha* samples from Nepal showed *Firmicutes* (80%) followed by *Actinobacteria* (20%); Darjeeling showed *Firmicutes* (100%); Sikkim showed *Firmicutes* (92%), and *Actinobacteria* (8%); Bhutan showed *Firmicutes* (100%). In starters from Arunachal Pradesh the variable distribution pattern in phyla level was observed. Samples of *paa* showed *Firmicutes* (80%), and *Proteobacteria* (20%), *pee* showed *Firmicutes* (67%), *Proteobacteria* (16%), and *Actinobacteria* (17%), and *phut* showed *Firmicutes* (75%), and *Proteobacteria* (25%). Similarly, phylum distribution in *phab* from Bhutan showed *Firmicutes* (57%) and *Proteobacteria* (43%). Based on results of the 16S rRNA gene sequencing, 15 different genera viz. *Leuconostoc, Enterococcus, Bacillus, Staphylococcus, Lactobacillus, Enterobacter, Klebsiella, Pseudomonas, Pediococcus, Stenotrophomonas, Kocuria, Brevibacterium, Lysinibacillus, Weissella*, and *Micrococcus* with 32 species from starters of the Eastern Himalayas were identified (Tables 3, 4). A wide diversity of bacteria (mainly LAB) was reported for the first time in traditionally prepared dry starters of the Eastern Himalayas (Table 5). The dominance of species of LAB was observed with 59% of total isolates in samples over non-LAB isolates (31%) (Figure 3). *Enterococcus durans, E. faecium, E. fecalis, E. hirae, E. lactis, Pediococcus acidilactici, P. pentosaceus, Lactobacillus plantarum* subsp. *plantarum, Lb. pentosus, Leuconostoc mesenteroides*, and *Weissella cibaria* were lactic acid bacterial species found in starter samples. *Enterococcus durans* (54.5%) was the most dominant species present in *marcha* samples from India (Darjeeling), whereas *Pediococcus pentocaseus* (5.8%) showed the lowest prevalence in *marcha* samples from Bhutan (Figure 4). LAB were found in all samples with highest occurrence in *marcha* samples of Darjeeling (91%). Non-LAB species were also recovered in many samples of starters, which were represented by *Bacillus subtilis* subsp. *inaquosorum, B. circulans, B. albus, B. cereus, B. nakamurai, B. nitratireducens, B. pseudomycoides, B. zhangzhouensis, Kocuria rosea, Staphylococcus hominis* subsp. *hominis, S. warneri, S. gallinarum, S. sciuri, Lysinibacillus boronitolerans, Brevibacterium frigoritolerans*, and *Micrococcus yunnanensis.* Interestingly, we detected few Gram-negative bacteria in some of the starter cultures from Arunachal Pradesh such as *Stenotrophomonas maltophilia* in *paa, Klebsiella pneumoniae* in *pee, Pseudomonas putida* in *phut*, and *Enterobacter hormaechei* subsp. *xiangfangensis*, and *E. hormaechei* subsp. *steigerwaltii* in some samples of *phab* from Bhutan (Table 5).

**TABLE 3:** Identification of LAB isolates from dry starters from the Eastern Himalayas based on 16S rRNA gene sequencing.

**Isolate code**	**Sample (Place)**	**Identity**	**Type species (% similarity)**	**GenBank Accession No.**	**Size (base pair)**
AKB6	*Marcha* (Darjeeling)	*Leuconostoc mesenteroides*	*Leuconostoc mesenteroides* ATCC 8293 (99.54%)	MK748250	1315
BPB18	*Marcha* (Bhutan)	*Enterococcus durans*	*Enterococcus durans* JCM 8725 16S (99.52%)	MK748251	1254
DMB4	*Marcha* (Darjeeling)	*Enterococcus durans*	*Enterococcus durans* JCM 8725 (99.55%)	MK748252	1325
SMB13	*Marcha* (Sikkim)	*Leuconostoc mesenteroides*	*Leuconostoc mesenteroides* ATCC 8293 (99.55%)	MK748253	1339
AKB3	*Marcha* (Darjeeling)	*Pediococcus acidilactici*	*Pediococcus acidilactici* DSM 20284 (99.62%)	MK748254	833
AOB14	*Pee* (Arunachal Pradesh)	*Enterococcus durans*	*Enterococcus durans* JCM 8725 (99.62%)	MK748255	1333
AOB15	*Pee* (Arunachal Pradesh)	*Enterococcus faecium*	*Enterococcus faecium* DSM 20477 (98.11%)	MK748256	1430
AOB25	*Phut* (Arunachal Pradesh)	*Enterococcus faecium*	*Enterococcus faecium* DSM 20477 (99.71%)	MK748258	1406
AOB4	*Paa* (Arunachal Pradesh)	*Enterococcus faecium*	*Enterococcus faecium* DSM 20477 (99.38%)	MK748259	1460
BPB11	*Marcha* (Bhutan)	*Enterococcus faecium*	*Enterococcus faecium* DSM 20477 (98.91%)	MK748260	1476
BPB31	*Phab* (Bhutan)	*Enterococcus faecium*	*Enterococcus faecium* DSM 20477 (99.28%)	MK748264	1390
BPB33	*Phab* (Bhutan)	*Enterococcus faecium*	*Enterococcus faecium* DSM 20477 (99.51%)	MK748265	1432
DMB11	*Marcha* (Darjeeling)	*Enterococcus durans*	*Enterococcus durans* JCM 8725 (99.78%)	MK748267	1342
DMB12	*Marcha* (Darjeeling)	*Pediococcus acidilactici*	*Pediococcus acidilactici* DSM 20284 (99.59%)	MK748268	1462
DMB6	*Marcha* (Darjeeling)	*Enterococcus durans*	*Enterococcus durans* JCM 8725 (99.38%)	MK748269	1443
MBV14	*Pee* (Arunachal Pradesh)	*Enterococcus durans*	*Enterococcus durans* JCM 8725 (99.86%)	MK748270	1436
SMB15	*Marcha* (Sikkim)	*Enterococcus faecium*	*Enterococcus faecium* DSM 20477 (99.86%)	MK748274	1447
SMB21	*Marcha* (Sikkim)	*Enterococcus faecium*	*Enterococcus faecium* DSM 20477 (99.64%)	MK748276	1400
SMB5	*Marcha* (Sikkim)	*Enterococcus faecium*	*Enterococcus faecium* DSM 20477 (99.78%)	MK748277	1391
SMB7	*Marcha* (Sikkim)	*Enterococcus durans*	*Enterococcus durans* JCM 8725 (98.71%)	MK748278	1158
AOB5	*Paa* (Arunachal Pradesh)	*Enterococcus faecalis*	*Enterococcus faecalis* ATCC 19433(T) (99.86%)	MK202997	1421
BPB13	*Marcha* (Bhutan)	*Pediococcus pentosaceus*	*Pediococcus pentosaceus* DSM 20336(T) (99.73%)	MK203008	1456
BPB21	*Phab* (Bhutan)	*Enterococcus durans*	*Enterococcus durans* NBRC 100479(T) (99.79%)	MK203010	1430
BPB4	*Marcha* (Bhutan)	*Enterococcus durans*	*Enterococcus durans* NBRC 100479(T) (99.65%)	MK203013	1437
DMB3	*Marcha* (Darjeeling)	*Enterococcus durans*	*Enterococcus durans* NBRC 100479(T) (99.72%)	MK203015	1441
AOB24	*Phut* (Arunachal Pradesh)	*Enterococcus hirae*	*Enterococcus hirae* ATCC 9790(T) (99.86%.)	MK202998	1411
DMB13	*Marcha* (Darjeeling)	*Enterococcus durans*	*Enterococcus durans* NBRC 100479(T) (99.58%)	MK203017	1433
DMB14	*Marcha* (Darjeeling)	*Pediococcus acidilactici*	*Pediococcus acidilactici* DSM 20284(T) (99.52%)	MK203018	1461
DMB11	*Marcha* (Darjeeling)	*Pediococcus acidilactici*	*Pediococcus acidilactici* DSM 20284(T) (99.66%)	MK203019	1456
DMB15	*Marcha* (Darjeeling)	*Enterococcus durans*	*Enterococcus durans* NBRC 100479(T) (99.72%)	MK203020	1437
NMB3	*Marcha* (Nepal)	*Lactobacillus pentosus*	*Lactobacillus pentosus* DSM 20314(T) (97.44%)	MK203022	1276
NMB8	*Marcha* (Nepal)	*Lactobacillus plantarum* subsp. *plantarum*	*Lactobacillus plantarum* subsp. *plantarum* ATCC 14917(T) (99.65%)	MK203024	1441
AOB26	*Phut* (Arunachal Pradesh)	*Enterococcus lactis*	*Enterococcus lactis* BT159 (T) (98.0%)	MK202999	1398
NMB7	*Marcha* (Nepal)	*Lactobacillus plantarum* subsp. *plantarum*	*Lactobacillus plantarum* subsp. *plantarum* ATCC 14917(T) (100%)	MK203027	1435
SMB9	*Marcha* (Sikkim)	*Weissella cibaria*	*Weissella cibaria* KACC 11862(T) (99.66%)	MK203028	1455
SMB13	*Marcha* (Sikkim)	*Pediococcus pentosaceus*	*Pediococcus pentosaceus* DSM 20336(T) (99.79%)	MK203029	1433
AOB2	*Paa* (Arunachal Pradesh)	*Enterococcus faecalis*	*Enterococcus faecalis* ATCC 19433(T) (99.79%).	MK203002	1420
AOB11	*Paa* (Arunachal Pradesh)	*Enterococcus faecalis*	*Enterococcus faecalis* ATCC 19433(T) (99.58%).	MK203003	1421
SMB11	*Marcha* (Sikkim)	*Enterococcus durans*	*Enterococcus durans* JCM 8725 (96.44%)	MK752677	1432
SMB3	*Marcha* (Sikkim)	*Enterococcus faecalis*	*Enterococcus faecalis* NBRC 100480 (97.42%)	MK752675	1123

*ATCC, American Type Cell Culture; JCM, Japan Collection of Microorganisms; DSM, Deutsche Sammlung von Mikroorganismen; MCCC, Microbial Culture Collection; NBRC, Biological Resource Centre, NITE; CCM, Czech collection of microorganisms; KACC, Korean Agricultural Culture Collection; YIM, Yunnan Institute of Microbiology; KCTC, Korean Collection for Type Cultures; NRRL, Agricultural Research Service Culture Collection.*

**TABLE 4:** Identification of non-LAB and Gram-negative bacteria from dry starters from the Eastern Himalayas based on 16S rRNA gene sequencing.

**Isolate code**	**Sample (Place)**	**Identity**	**Type species (% similarity)**	**GenBank Accession No.**	**Size (base pair)**
AOB48	*Phut* (Arunachal Pradesh)	*Pseudomonas putida*	*Pseudomonas putida* KT2440 (99.85%)	MK203004	1379
AOB18	*Pee* (Arunachal Pradesh)	*Klebsiella pneumoniae*	*Klebsiella pneumoniae* DSM 30104 (99.3%)	MK748257	1439
BPB23	*Phab* (Bhutan)	*Enterobacter hormaechei* subsp. *xiangfangensis*	*Enterobacter hormaechei subsp. Xiangfangensis* 10−17 (99.58%)	MK748261	1431
BPB27	*Phab* (Bhutan)	*Enterobacter hormaechei* subsp. *Xiangfangensis*	*Enterobacter hormaechei* subsp. *Xiangfangensis* 10−17 (98.88%)	MK748263	1446
BPB26	*Phab* (Bhutan)	*Enterobacter hormaechei* subsp. *steigerwaltii*	*Enterobacter hormaechei* subsp. *Steigerwaltii* DSM 16691(T) (99.23%)	MK203011	1422
AOB9	*Paa* (Arunachal Pradesh	*Stenotrophomonas maltophilia*	*Stenotrophomonas maltophilia* MTCC 434(T) (99.79%)	MK203000	1416
NMB10	*Marcha* (Nepal)	*Bacillus zhangzhouensis*	*Bacillus zhangzhouensis* DW5−4(T) (99.58%)	MK203023	1432
NMB23	*Marcha* (Nepal)	*Staphylococcus xylosus*	*Staphylococcus xylosus* CCM 2738(T) (99.86%)	MK203021	1426
BPB24	*Phab* (Bhutan)	*Bacillus albus*	*Bacillus albus* MCCC 1A02146 (99.02%)	MK748262	1437
BPB8	*Marcha* (Bhutan)	*Bacillus circulans*	*Bacillus circulans* NBRC 13626 (98.64%)	MK748266	1412
NMB11	*Marcha* (Nepal)	*Bacillus cereus*	*Bacillus cereus* ATCC 14579 (100%)	MK748271	1460
NMB12	*Marcha* (Nepal)	*Brevibacterium frigoritolerans*	*Brevibacterium frigoritolerans* DSM 8801 (99.72%)	MK748272	1426
NMB13	*Marcha* (Nepal)	*Brevibacterium frigoritolerans*	*Brevibacterium frigoritolerans* DSM 8801 (100%)	MK748273	1388
SMB19	*Marcha* (Sikkim)	*Lysinibacillus boronitolerans*	*Lysinibacillus boronitolerans* NBRC 103108 (99.59%)	MK748275	1220
BPB1	*Marcha* (Bhutan)	*Staphylococcus warneri*	*Staphylococcus warneri* ATCC 27836(T) (99.72%)	MK203006	1437
BPB10	*Marcha* (Bhutan)	*Staphylococcus warneri*	*Staphylococcus warneri* ATCC 27836(T) (99.79%)	MK203007	1432
BPB17	*Marcha* (Bhutan)	*Staphylococcus warneri*	*Staphylococcus warneri* ATCC 27836(T) (99.72%)	MK203009	1429
BPB3	*Marcha* (Bhutan)	*Staphylococcus warneri*	*Staphylococcus warneri* ATCC 27836(T) (98.92%)	MK203012	1490
BPB7	*Marcha* (Bhutan)	*Bacillus nitratireducens*	*Bacillus nitratireducens* 4049(T) (99.36%)	MK203014	1404
DMB5	*Marcha* (Darjeeling)	*Staphylococcus hominis* subsp. *hominis*	*Staphylococcus hominis* subsp. *hominis* DSM 20328(T) (99.93%)	MK203016	1425
NMB20	*Marcha* (Nepal)	*Staphylococcus gallinarum*	*Staphylococcus gallinarum* ATCC 35539(T) (99.86%)	MK203025	1437
NMB22	*Marcha* (Nepal)	*Staphylococcus sciuri*	*Staphylococcus sciuri* DSM 20345(T) (99.65%)	MK203026	1439
SMB22	*Marcha* (Sikkim)	*Micrococcus yunnanensis*	*Micrococcus yunnanensis* YIM 65004(T) (99.64%)	MK203030	1379
SMB1	*Marcha* (Sikkim)	*Bacillus subtilis* subsp. *inaquosorum*	*Bacillus subtilis* subsp. *Inaquosorum* KCTC 13429(T) (99.65%)	MK203031	1425
SMB8	*Marcha* (Sikkim)	*Bacillus pseudomycoides*	*Bacillus pseudomycoides* AFS041167 (99.93%)	MK203032	1407
AOB19	*Pee* (Arunachal Pradesh)	*Kocuria rosea*	*Kocuria rosea* DSM 20447(T) (99.79%.)	MK203001	1399
AOB20	*Pee* (Arunachal Pradesh)	*Bacillus subtilis* subsp. *inaquosorum*	*Bacillus subtilis* subsp. *Inaquosorum* KCTC 13429(T) (99.79%)	MK203005	1431
SMB14	*Marcha* (Sikkim)	*Bacillus nakamurai*	*Bacillus nakamurai* NRRL B-41091 (96.65%)	MK752676	1103

**TABLE 5:** Bacterial diversity in dry starters from the Eastern Himalayas.

**Country/place**	**Starter**	**Bacterial species**	
Nepal	*Marcha*	LAB:	*Lactobacillus pentosus, Lb. plantarum* subsp. *plantarum*
		Non-LAB:	*Bacillus cereus, B. zhangzhouensis, Brevibacterium frigoritolerans, Staphylococcus xylosus, S. gallinarum, S. sciuri*
		Gram-ve bacteria:	NR
India (Darjeeling hills)	*Marcha*	LAB:	*Enterococcus durans, Pediococcus acidilactici, Leuconostoc mesenteroides*
		Non-LAB:	*Staphylococcus hominis* subsp. *hominis*
		Gram-ve bacteria:	NR
India (Sikkim)	*Marcha*	LAB:	*Pediococcus pentosaceus, Leuconostoc mesenteroides, Enterococcus faecium, E. faecalis, E. durans, Weissella cibaria*
		Non-LAB:	*Lysinibacillus boronitolerans, Micrococcus yunnanensis, Bacillus subtilis* subsp. *inaquosorum, B. pseudomycoides, B. nakamurai*
		Gram-ve bacteria:	NR
India (Arunachal Pradesh)	*Paa*	LAB:	*Enterococcus faecalis, E. faecium*
		Non-LAB:	NR
		Gram-ve bacteria:	*Stenotrophomonas maltophilia*
	*Pee*	LAB:	*Enterococcus faecalis, E. durans, E. faecium*
		Non-LAB:	*Kocuria rosea, Bacillus subtilis* subsp. *inaquosorum*
		Gram-ve bacteria:	*Klebsiella pneumoniae*
	*Phut*	LAB:	*Enterococcus hirae, E. lactis, E. faecium*
		Non-LAB:	NR
		Gram-ve bacteria:	*Pseudomonas putida*
Bhutan	*Marcha*	LAB:	*Pediococcus pentosaceus, Enterococcus durans, E. faecium*
		Non-LAB:	*Staphylococcus warneri, Bacillus nitratireducens, B. circulans*
		Gram-ve bacteria:	NR
	*Phab*	LAB:	*Enterococcus durans, E. faecium*
		Non-LAB:	*Bacillus albus*
		Gram-ve bacteria:	*Enterobacter hormaechei* subsp. *xiangfangensis, Enterobacter hormaechei* subsp. *steigerwaltii*

*LAB, lactic acid bacteria; NR, not recovered.*

Diversity indexes of bacterial communities of different starter cultures were characterized by the Shannon diversity index *H*, the Simpson’s index, and the Dominance and Chao1 index (Table 6). The Shannon diversity index *H* for evaluating bacterial diversity recorded highest in *marcha* from Sikkim (*H*:2.305) and lowest in *marcha* from Darjeeling (*H*:1.121). Simpson’s diversity index (1-*D*) values were 0.8878, 0.8711, 0.86, and 0.8374 for starters from Sikkim, Arunachal Pradesh, Nepal, and Bhutan, respectively. An estimation of species richness based on abundance was shown by the Chao 1 index. The dominance *D*-values were recorded as being highest for *marcha* samples from Darjeeling and lowest for *marcha* samples from Sikkim.

**TABLE 6:** Diversity indices of different dry starters from the Eastern Himalayas.

**Country/Region**	**Diversity indices**			
	
	**Simpson’s index (1-*D*)**	**Shannon’s index (*H*)**	**Dominance (*D*)**	**Chao-1**
Nepal	0.86	2.025	0.14	13
India (Darjeeling hills)	0.6116	1.121	0.3884	5
India (Sikkim)	0.8878	2.305	0.1122	29
India (Arunachal Pradesh)	0.8711	2.176	0.1289	20.5
Bhutan	0.8374	1.925	0.1626	14

## Discussion

In this study five types of traditionally prepared dry starters (*marcha, pha, paa, pee*, and *phut*) were collected from different regions of the Eastern Himalayas, and they were analyzed for microbial load, pH, and moisture. The average bacterial population of all samples was 10^8^ cfu/g, which was not reported earlier except for *marcha* from the Darjeeling hills and Sikkim (Tamang and Sarkar, 1995; Tsuyoshi et al., 2005; Tamang et al., 2007). The bacterial load of *marcha* from Sikkim was 10^6^ to 10^8^ cfu/g (Table 1), which was almost the same as that of populations of yeasts and filamentous molds in *marcha* from Sikkim (Tsuyoshi et al., 2005). This shows that bacterial populations in traditionally prepared starters of the Eastern Himalayas may have co-existed equally with filamentous molds and yeasts (Hesseltine et al., 1988; Zheng et al., 2015). The moisture content of all starters was low due to the sun-drying process that followed immediately after fermentation, the step necessary to maintain the potency of traditionally prepared starters to be able to be stored in a dry place at room temperature for future use. The pH of all samples was mildly acidic, which may be due to the dominance of LAB (∼10^8^ cfu/g) in dry starters (Tamang and Sarkar, 1995).

First, we phenotypically characterized all 201 bacterial strains isolated from samples of *marcha, paa, pee, phut*, and *phab* and presumptively identified four genera of LAB- *Enterococcus, Pediococcus, Leuconostoc*, and *Lactobacillus*, two genera of non-LAB-*Bacillus* and *Staphylococcus*, and two Gram-negative bacterial genera, *Enterobacter* and *Citrobacter.* We grouped 201 isolates into 68 representative bacterial strains on the basis of phenotypic and biochemical tests for confirmation of their identity and assigned the taxonomical nomenclature by using 16S rRNA gene sequencing. In our study, we found a dominance of phylum *Firmicutes* (85%) over *Proteobacteria* (9%) and *Actinobacteria* (6%) in starters from the Eastern Himalayas. *Firmicutes* was also reported as the major abundant phylum in *daqu*, a starter for Chinese strongly flavored liquor (Zou et al., 2018; He et al., 2019), and in *nuruk*, a starter from Korean used to produce *makgeolli*, a Korean alcoholic beverage (Jung et al., 2012). The sequence data based on a constructed phylogenetic tree revealed a dominance of LAB (59%) with five different genera and 11 species represented by *Enterococcus durans, E. faecium, E. fecalis, E. hirae, E. lactis, Pediococcus acidilactici, P. pentosaceus, Lactobacillus plantarum* subsp. *plantarum, Lb. pentosus, Leuconostoc mesenteroides*, and *Weissella cibaria*. Only two genera of LAB represented by *Pediococcus pentosaceus* and *Lactobacillus brevis* were reported earlier from *marcha* samples from Sikkim and the Darjeeling hills (Tamang and Sarkar, 1995; Tamang et al., 2007). However, in this study we found a wide diversity of LAB in samples of *marcha* collected from the Darjeeling hills and Sikkim in India, which included *Pediococcus pentosaceus, P. acidilactici, Enterococcus faecium, E. durans, E. faecalis, Leuconostoc mesenteroides*, and *Weissella cibaria*, whereas, *Lactobacillus pentosus* and *Lb. plantarum* subsp. *plantarum* were found only in *marcha* samples from Nepal. Variations in altitude and other geographical factors may affect the composition of microbiota in dry starters (Jeyaram et al., 2011; Lv et al., 2012). Traditional methods of preparation of *marcha, phab, paa, pee*, and *phut* are more or less similar except for some variations that were observed in the use of substrates, such as rice for *marcha, phut, paa*, and *pee*, and maize-rice husk for *phab* from Bhutan, and also wrapping materials for fermenting substrates such as fern leaves (*Glaphylopteriolopsis erubeseens*) for *marcha* preparation, dry paddy straws for *phab*, and locally available plant leaves for the preparation of *paa, pee*, and *phut*. Bacterial diversity in dry starters from the Eastern Himalayas may be influenced by hygienic conditions, quality of cereal substrates, wrapping materials, and sources of natural or tap water during traditional methods of preparation (Peter-Ikechukwu et al., 2016; Gonelimali et al., 2018; Sha et al., 2019).

The bacterial profile of *marcha* from Nepal and Bhutan, *paa, pee*, and *phut* of Arunachal Pradesh, and *phab* from Bhutan has been reported for the first time in our study. A similar type of dry starter for Assam in North East India called *xaj-pitha* also contained several species of LAB such as *Lactobacillus plantarum, Lb. brevis, Weissella cibaria, W. paramesenteroides, W. confusa, Lactococcus lactis, Lactobacillus casei group, Leuconostoc lactis, Leuconostoc pseudomesenteroides, Pediococcus pentosaceus, Lactococcus garvieae*, and *Enterococcus* sp. (Bora et al., 2016). Thanh et al. (2008) reported many species of LAB in Vietnamese *banh men*, which included *Pediococcus pentosaceus, Lactobacillus plantarum, Lb. brevis, Lb. fermentum, Lb. agilis, W. confusa, W. paramesenteroides*, and *Lactococcus lactis*. *Enterococcus faecium, Lactobacillus plantarum, Leuconostoc mesenteroides, Pediococcus acidilactici, P. pentosaceus, Weissella paramesenteroides*, and *W. cibaria*, were reported in *nuruk* from Korea (Hoon et al., 2013). Several species of LAB in Cambodian *dombea* were also reported: *Weissella cibaria, Lactobacillus plantarum, Lactococcus lactis, Pediococcus pentosaceus*, and *Enterococcus durans* (Ly et al., 2018). This indicates that species of LAB predominate the microbial composition of traditionally prepared dry starters in Asia, including the Eastern Himalayas. LAB have been considered as favorable bacteria in cereal-based beverages due their ability to improve protein digestibility, enhance organoleptic quality, and increase nutritional bioavailability (Luana et al., 2014). Species of *Weissella, Lactobacillus, Lactococcus, Leuconostoc, Pediococcus*, and *Enterococcus* are known for flavor development, the production of organic acids, and antimicrobial activities in Chinese *daqu* used for liquor production (Gou et al., 2015). *Enterococcus* sp. has been reported to produce enterocins, which play a major role in preventing the growth of foodborne and spoilage-causing pathogens (Javed et al., 2011).

Non-LAB species formed the next abundant group (32%) in starters from the Eastern Himalayas with the dominance of *Bacillus* spp. The abundance of *Bacillus* sp. may be due to its ability to survive in environments with low moisture and high temperature (Nuding et al., 2017). Also, the *Bacillus* species are important sources of amylase and protease enzymes, which are involved in saccharification and flavor production (Beaumont, 2002). A dominance of *Bacillus* sp. was also reported in *daqu* from China (Wang et al., 2008; Zheng et al., 2012) and *banh men* from Vietnam (Thanh et al., 2008). The next most abundant bacterium was *Staphylococcus* spp., found in the Himalayan starters, which secretes amylase (Li et al., 2014) and protease in Chinese *daqu* (Yang et al., 2017) and also produces lipases for the production of esters for flavor (Talon et al., 1996); thus, this group of bacteria probably plays a major role in the flavor enhancement of the final product. The prevalence of phylum *Actinobacteria* in some starters of the Eastern Himalayas was only 6%, represented by *Kocuria rosea*, *Micrococcus yunnanensis*, and *Brevibacterium frigoritolerans*. The presence of *Actinobacteria* has been reported in Chinese *daqu* (Zou et al., 2018) and Indian *marcha* and *thiat* (Sha et al., 2017).

Few species of opportunist pathogens and environmental contaminants such as *Micrococcus*, *Stenotrophomonas*, *Enterobacter*, *Klebsiella*, and *Pseudomonas* were detected, and they were found only in samples of *paa, pee*, and *phut* from Arunachal Pradesh, and *phab* from Bhutan. However, both the prevalence and populations of these contaminants were low and it is presumed that these organisms might have contaminated the samples during the traditional method of preparation from substrates, herbs, water, utensils, wrapping materials, etc., Gram-negative bacteria were not detected in any samples of *marcha* collected from Nepal, India, or Bhutan. In our previous study on *marcha*, no Gram-negative bacteria were found at the genus level, and this was discovered through an analysis using a high-throughput sequencing method (Sha et al., 2019). Although most of these bacteria are opportunists and probable foodborne pathogens, some of them, such as *Enterobacter* sp., are involved in the production of amylases and lipases and also the formation of flavor in *daqu* (Li et al., 2015). The presence of LAB inhibits the growth of pathogenic and spoilage microorganisms in foods (Cizeikiene et al., 2013; Castellano et al., 2017) and produces flavor compounds (Mukisa et al., 2017).

A diversity index, or phylogenetic metric, is a quantitative measure to show phylogenetic relations within different species in a community (Birtel et al., 2015). We characterized diversity indexes of the bacterial community present in starters from the Eastern Himalayas by using the Shannon diversity index *H*, Simpson’s index, and Dominance and Chao1 index (Table 6). The Shannon diversity index *H* for evaluating bacterial diversity was recorded as being highest in *marcha* from Sikkim (*H*:2.305) and lowest in *marcha* from Darjeeling (*H*:1.121), indicating a higher bacterial diversity in *marcha* from Sikkim as compared to other starters. The Simpson’s diversity index (1-*D*) index, which considers both the number of species as well as the relative abundance of each species for evaluating diversity, showed the highest values for *marcha* from Sikkim. The dominance *D*-values were recorded as being highest for *marcha* samples from Darjeeling and lowest for *marcha* samples from Sikkim, which supports the above inference regarding bacterial diversity. The dominance *D*-value ranged between 0–1, where the value 0 indicated that all taxa were equally present and value 1 indicated the dominance of one taxon over the whole community (Wagner et al., 2018). Thus, the values near zero indicate a highly diverse ecosystem and values near 1 indicate a less diverse or homogenous ecosystem (Lv et al., 2012). Hence, the phylogenetic matric of the bacterial community present in dry starters from the Eastern Himalayas showed high diversity within the community. The Eastern Himalayas are known for their rich floral and faunal diversity within a wide ecosystem (Chettri et al., 2010). Our findings thus highlight the richness of microbial diversity in the food ecosystem of the Eastern Himalayas.

The microbial communities and their interactions in starters are extremely important for proper fermentation, which may determine the productivity and flavor quality of the final alcoholic beverage (Cai et al., 2018). There has been an increasing amount of concern regarding the safety of fermented beverages due to the presence of ethyl carbamate, which is considered to be carcinogenic (Ryu et al., 2015), biogenic amines (Liu et al., 2016), mycotoxin (Sivamaruthi et al., 2018), and contamination by opportunistic microbial pathogens (Hong et al., 2016). All these considerations mandate a deep understanding of the microbial community in starters. Also, the profile of native microbiota in these starters opens a possibility of finding novel strain(s) with functional properties for industrial purposes. This study also records the bacterial diversity of *phab* from Bhutan, which is found to be produced rarely by a few ethnic people of Bhutan. This is probably due to their preference for commercial *marcha*, similar to *phab*, which is sold in local markets. Bacteria present in traditionally prepared dry starters have no amylolytic activities (Thapa and Tamang, 2004); however, they may contribute to the acidification of fermenting substrates and impart flavor with a mildly acidic and sour taste to traditional alcoholic beverages (*kodo ko jaanr, opo, apong*, and *themsing*) preferred by the Himalayan people (Thapa and Tamang, 2006; Tamang et al., 2007).

## Conclusion

Information on the microbial composition of traditionally prepared dry starters of the Eastern Himalayan regions of India, Nepal, and Bhutan viz. *phab, paa, pee*, and *phut*, was unknown except for *marcha* from Sikkim in India. These traditional starters are used by the Himalayan people to ferment cereals into various alcoholic beverages for home consumption. The main objective of this study was to profile and assign the taxonomical identity of bacteria isolated from these traditional starters of the Eastern Himalayas based on 16S rRNA sequencing. *Firmicutes* was the most dominant phylum in all starters and was represented by several genera and species of LAB and also by some non-LAB. Interestingly our study showed high diversity within the bacterial community in traditionally prepared starters of the Eastern Himalayas, which may supplement the richness of microbial conservation in the food ecosystem of the regions. Besides diversity, some bacteria isolated from these traditional starters may have commercial and industrial importance. This is the first report on the bacterial diversity of dry starters of the Eastern Himalayas by Sanger sequencing.

## Data Availability Statement

The datasets generated for this study can be found in the sequences retrieved from the 16S rRNA sequencing were deposited at GenBank-NCBI under the nucleotide accession number: MK748250-MK748278, MK202997-MK203032, and MK752675-MK752677.

## Author Contributions

PP performed the majority of the experiments. JT supervised the experiments and finalized the manuscript.

## Conflict of Interest

The authors declare that the research was conducted in the absence of any commercial or financial relationships that could be construed as a potential conflict of interest.
